# Potential of Chemical and Physical Enhancers for Transungual Delivery of Amorolfine Hydrochloride

**DOI:** 10.3390/ma12071028

**Published:** 2019-03-28

**Authors:** Indrė Šveikauskaitė, Alius Pockevičius, Vitalis Briedis

**Affiliations:** 1 Department of Clinical Pharmacy, Faculty of Pharmacy, Lithuanian University of Health Sciences, Sukilėlių pr. 13, 50161 Kaunas, Lithuania; vitalis.briedis@lsmuni.lt; 2 Department of Veterinary Pathobiology, Lithuanian University of Health Sciences, Tilžės g. 18, 50161 Kaunas, Lithuania; alius.pockevicius@lsmuni.lt

**Keywords:** transungual delivery, polymeric nail lacquers, amorolfine hydrochloride, chemical enhancement, fractional laser, onychomycosis

## Abstract

Topical monotherapy of nail infection is limited by poor drug permeability into the human nail plate. Numerous substances and methods are applied to improve the antifungal agent delivery across the nail plate. This work aimed to evaluate the effect of chemical and physical enhancers on the accumulation and permeation of amorolfine hydrochloride through human nail clippings. Polymeric nail lacquers with Eudragit E100 were developed as a potentially suitable delivery system for amorolfine hydrochloride. Incorporating thioglycolic acid and urea into formulations provided increased accumulation of antifungal agent in nail layers of up to 100% and 57%, respectively. Structural changes of nail barrier, induced by fractional CO_2_ laser, were visualized by microscopy. The permeation of amorolfine hydrochloride through the nail increased twofold when thioglycolic acid-containing formulation was applied and the nail was pretreated with a fractional CO_2_ laser. The results suggest that this novel combination of enhancers has the potential to be an effective option for topical drug delivery through the nail, and increased the efficacy of treatment.

## 1. Introduction

A human nail is composed of the nail plate (thin structure of approximately 25 layers of keratinocytes with keratin filaments matrix) and four epithelial tissues: nail matrix, hyponychium, nail bed, and perionychium [1]. Onychomycosis is a common nail disease which affects up to 14% of the population [2]. The current treatment of onychomycosis includes systemic therapies, topically applied products, and surgical interventions [3]. Systemic treatment of onychomycosis presents safety issues due to possible drug–drug interactions and hepatotoxicity [3,4]. Topical therapy of onychomycosis is advantageous due to its localized effect and that delivery of the drug to the infected nail is direct. Topical therapy offers decreased systemic side effects, however, the efficacy of such therapy depends on achieving effective concentrations of antifungal agents at the infection site [5,6]. 

The primary target tissues for fungal infection therapy are the nail plate and the nail bed [7]. The presence of keratin in the structure of the nail results in rigidity of the structure, thus causing decreased permeability of the drugs, and their low concentrations at the target area [8]. Drug affinity to the keratin network decreases the permeability of the nail and can cause drug molecule binding which can reduce antifungal activity. The presence of such interactions requires higher doses to ensure the efficacy of treatment [7]. This effect was observed during in vitro studies using amorolfine hydrochloride as an active antifungal agent. It has been demonstrated that the amorolfine hydrochloride minimum inhibitory concentration (MIC) is lower than the minimum fungicidal concentration (MFC) in the nail (Nail-MFC)—respectively 0.25 and 2 µg/mL [9]. Amorolfine hydrochloride has demonstrated stronger cidal action and appears to be the most effective topical antifungal agent in onychomycosis treatment on the basis of MFC when compared to other antifungals [10]. As evident from results of in vitro experiments using the classical standard in vitro media, water or phosphate buffer, increased concentrations and/or longer incubation times of amorolfine hydrochloride are necessary for effectively inactivating *Trichophyton rubrum* [9]. Therefore, the development and evaluation of nail lacquer formulations can offer numerous advantages over systemic antifungal therapy.

Enhanced intraungual drug delivery could be achieved by the application of mechanical, chemical, or physical methods. The selection of chemical enhancers and their incorporation into dosage forms is considered as an efficient approach to achieving increased ungual penetration and permeability [11]. Typically, transungual permeation enhancers induce nail plate hydration and swelling. This results in increased nail plate porosity and diffusion [7]. Molecules containing sulfhydryl groups are considered to be efficient intraungual penetration enhancers. Such compounds usually demonstrate the ability to cause nail plate swelling and increase the porosity of the nail via disruption of disulfide bonds of keratin molecules. These changes are irreversible, and can result in increased drug penetration into the nail [12,13]. Keratolytic agents (e.g., urea) also cause nail plate softening, but via keratin denaturation [7]. Controversial results were demonstrated by application of organic solvents (e.g., ethanol, isopropanol, or isopropyl myristate), which are known as transdermal permeation enhancers. Organic solvents should enhance drug penetration by modifying the hydration state of nail [14], though this effect is hardly possible because of the low lipid content in the nail plate and the dehydrating/deswelling effect of these solvents [15]. 

Enhancement of physical drug penetration is considered as an efficient alternative approach to increase transungual delivery. Compared with other physical enhancement techniques, laser application presents minimal invasiveness which may enhance patient compliance. Laser disrupts the nail barrier by creating microchannels on the nail surface. This effect leads to an increased contact area between the topical formulation and the nail, thus enhancing drug penetration [16]. 

The development and application of an appropriate in vitro model system could provide a means to reliably evaluate penetration/permeation enhancers. It is of critical importance to choose adequate membranes for the evaluation of transungual formulations, as human nail plates are usually unavailable in the required quantities and are difficult to standardize. Nail clippings could be considered as a possible alternative as their availability is not limited, but they are of curved shape, hard, and relatively smaller in size. Highly sensitive analytical techniques and a long duration of the testing procedure are needed to achieve detectible drug amounts in the tested biological matrix [7].

A possible approach to mimic the human nail plate is to use animal nail of similar porosity and swelling capacity. It was confirmed that bovine hoof membranes are suitable for transungual permeation studies. Certain differences have to be taken into consideration when designing transungual penetration studies. Bovine hoof membranes demonstrate higher swelling capacities compared to human nails (~38% vs. ~25%) [7] and a higher porosity of the keratin network [17]. Therefore, it could be expected that higher permeation of the drugs could be determined when bovine hoof membranes are applied [7,18]. However, drug affinity to bovine hoof keratin is considered to be similar to human nail keratin [7], and such a nail model is still often used in transungual penetration studies. 

In this study, we used chemical enhancers and fractional CO_2_ laser to treat bovine hoof membranes (for use as an in vitro model of human nail) and human nail clippings for evaluation of enhanced transungual delivery of amorolfine hydrochloride from nail lacquers in vitro. Also for the first time, the combined effect of CO_2_ laser and chemical enhancers on the permeation of amorolfine hydrochloride into and across human nail was evaluated. 

## 2. Materials and Methods

### 2.1. Materials

Amorolfine hydrochloride was purchased from ChemicalPoint (Deisenhofer, Germany). Ethanol 96% (Stumbras, Lithuania) was used as a solvent, diethylene glycol monoethyl ether (Transcutol P) (Gattefosse, France) as a plasticizer, while film-forming polymer amino methacrylate copolymer (Eudragit E100) was kindly gifted by Evonik Industries AG (Essen, Germany). Salicylic acid was obtained from Alfa Aesar (Germany); glycerol from Applichem (Germany). 1,2-propanediol, polyethylene glycol 400; polyethylene glycol 1500; and urea were purchased from Roth (Karlsruhe, Germany) and used as chemical enhancers. Tween 60, Tween 40, citric acid monohydrate, sodium carbonate, acetone, benzoic acid, methanol, and ethyl acetate were used as enhancers, and obtained from Sigma-Aldrich Chemie GmbH (Steinheim, Germany). Triacetin was kindly supplied by Lanxess (Germany), while thioglycolic acid was kindly gifted by Merck Group (Darmstadt, Germany). Acetonitrile and trifluoroacetic acid for chromatographic analysis were purchased from Sigma-Aldrich Chemie GmbH (Steinheim, Germany).

### 2.2. Methods

#### 2.2.1. Preparation of Hooves and Nails

Hooves were taken from freshly slaughtered cattle 2–3 years of age, stripped of adhering cartilaginous, connective tissue, and kept in distilled water for 72 h [18]). Sections, approximately 60 µm thick, were taken from the bottom of the hoof using a cryotome (Thermo Scientific Cryotome FSE). Prepared hoof membranes were kept at −20 °C and moved to room temperature 5 h before use in penetration studies. 

Nail clippings were obtained from healthy human volunteers (male and female, aged 25–40 years) using nail clippers. Nail clippings were washed with phosphate buffer (pH 7.4) and wiped with filter paper. 

#### 2.2.2. Selection and Evaluation of Chemical Enhancers

**Figure 1:**
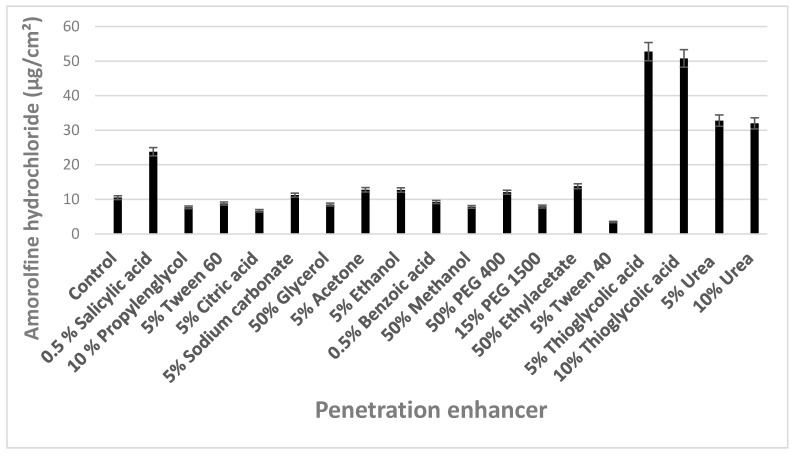
Quantity of amorolfine hydrochloride absorbed by hoof lamella after 24 h pretreatment in solutions of penetration enhancers. Control—acidified water (pH 3).

The desired concentrations of enhancers (listed in Figure 1) were incorporated in acidified water, and the final pH was adjusted to 3, for maximum solubility of amorolfine hydrochloride. The concentrations of applied enhancers were chosen referring either to their solubility at pH 3 or to the published scientific data.

Bovine hoof membranes were placed into the above solutions and kept at 32 °C for 24 h. After washing and drying, hoof membranes were weighed, placed into amorolfine hydrochloride solution (500 µg/mL) and incubated for 24 h at 35 °C. Membranes were washed, and active substance was extracted with methanol by sonication for 30 min and analyzed for amorolfine hydrochloride content. The controls were carried out using a “drug only” formulation containing 500 µg/mL amorolfine hydrochloride solution in acidified (pH 3) water. 

#### 2.2.3. Physical Enhancement

Candela CO_2_RE laser (Syneron Candela, CA, USA) was used to disrupt the nail barrier to enhance transungual delivery of amorolfine hydrochloride. Nail clippings were treated by the laser at different energy levels: “fusion” (50–70 mJ energy) and “deep” (60–80 mJ energy) before nail lacquer application. Structural changes of nail barrier were visualized using optical microscope Optika B-353FL (Optika, Ponteranica, Italy). 

#### 2.2.4. Preparation and Evaluation of Experimental Formulations

The experimental nail lacquers were formulated by dissolving the amount of amorolfine HCl in a lacquer base, containing ethanol (96%), Transcutol P, film-forming polymer Eudragit E100, and an appropriate chemical enhancer. 

Quality of nail lacquers (compositions are listed in Table 1) was evaluated by measuring drying time, water resistance, and amorolfine hydrochloride release. 

Drying time was evaluated by applying a liquid film of the experimental sample on a glass plate and the time until obtaining a dry-to-touch state was determined. The water resistance test was performed by applying a nail lacquer onto a glass slide, allowing it to dry, then immersing it into distilled water for 7 days [19,20]. 

Amorolfine hydrochloride release testing was carried out at 32 °C by measuring drug quantity diffusion through 1 cm^2^ Cuprophan dialysis membranes (MWCO—10,000 Da). For each of the different experimental formulations, a 50 µL aliquot of lacquer was uniformly applied to the surface of the membrane and left for 4 h to form a film. Dry films were protected from external factors by aluminum foil cover [21]. 

Amorolfine hydrochloride solubility in PBS at 32 °C was determined to be 3749 µg/mL and PBS (15 mL) was used as the acceptor phase for establishing *sink* conditions. At predefined time points, 1 mL of acceptor medium was taken, and the same amount of fresh medium was added to maintain a constant volume of acceptor liquid. The amount of released amorolfine hydrochloride was determined by UPLC chromatography at 218 nm wavelength in triplicates. 

#### 2.2.5. Nail Penetration Studies

Amorolfine hydrochloride transungual penetration was studied by determining amorolfine hydrochloride quantity in human nail clippings. The lacquer sample was applied in three layers, allowing complete drying until constant weight was achieved. The ventral sides of the nail clippings were placed on small wetted cotton balls (that functioned as an acceptor compartment and supporting nail bed). The moisture to the nail plate was provided by cotton balls [22]. The average surface area of nail clippings was 0.45 cm^2^.

Clippings with wetted cotton balls were wrapped in aluminum foil and incubated at 32 °C for 24 h. Afterwards, nails were cleaned, washed, and dried. The weight and thickness of each nail was measured. All clippings were transferred to Eppendorf tubes and extracted with 1 mL of methanol at 32 °C for 24 h. Amorolfine hydrochloride in cotton balls was determined by extracting with methanol for 24 h at 32 °C, and was performed twice. The amount of amorolfine hydrochloride in all samples was determined by UPLC chromatography. 

#### 2.2.6. Analytical Procedures

Amorolfine hydrochloride content was quantified by liquid chromatography using Acquity UPLC H-Class chromatography system (Waters, Milford, MA, USA) equipped with DAD (Waters, Milford, MA, USA), performing detection at 218 nm. Separation was performed on Acquity UPLC BEH C18 (130 Å, 1.7 µm, 2.1 mm × 50 mm, Waters, Milford, MA, USA) column. The mobile phase was delivered in a linear elution gradient from 70% to 40% of solvent A (acetonitrile) in B (0.1% (v/v) trifluoroacetic acid in ultrapure water) for 5 min; the injection volume was 1 µL, flow rate was 0.7 mL/min, and the column temperature was 30 °C. A standard calibration curve was built up by using standard solutions (2.4–458.65 µg/mL). The developed UPLC method was validated in terms of precision, accuracy, and linearity according to ICH guidelines [23]. Assay method precision was carried at LOQ level and determined using seven independent test solutions. The %RSD was within the acceptable limit of 2%. Linear calibration plots were obtained at seven concentration levels in triplicate and linearity confirmed for the range 2.4–458.65 µg/mL (*R*^2^ = 0.9999). The results showed excellent correlation between the peak area and concentration. The accuracy of the assay method was evaluated with the recovery of the standards from samples in triplicate. The recovery of the investigated components ranged from 96% to 99%, and their RSD values were less than 5%, indicating good reliability and accuracy of the method. The LOD was 0.6 µg/mL, and LOQ was 1.9 µg/mL for amorolfine UPLC method. 

#### 2.2.7. Statistical Analysis

Results are presented as means ± SD. Spearman’s rank coefficient was used for correlation analysis. Statistically significant difference was determined when the value of *p* < 0.05. Results were calculated using IBM SPSS Statistics for Windows Version 19.0(IBM Corporation, Armonk, NY) and Microsoft Office Excel 2015. 

## 3. Results and Discussion

### 3.1. Evaluation of Chemical Enhancers for Transungual Drug Delivery

The excipients of the lacquers and their quantitative composition can have a crucial effect of drug release from the dried film of lacquer and its penetration into the nail. Screening of potential transungual chemical enhancers was performed applying general procedures, described by Murthy et al. [24]. Selection of chemical enhancers was performed by using bovine hoof membranes as a nail model. Monti et al. [25] validated bovine hoof membranes as a model for infected human toenails. Thus, bovine hoof membranes have been used as a model for human nail plates, which are less available for such permeation studies [18]. The hoof membranes were immersed in enhancer solutions for 24 h to allow enough time for any possible structural changes to occur. The amount of amorolfine hydrochloride that penetrated into the biomatrix depended on the changes of the nail structure, caused by the enhancers. 

Significant enhancement of amorolfine hydrochloride penetration into the hoof lamella was determined only after the bovine hooves were pretreated with 0.5% salicylic acid, and 5% and 10% of either urea or thioglycolic acid (Figure 1). Pretreatment of the nail with 5% Tween 40 solution caused a statistically significant decrease in drug penetration. This effect could be related to the presence of several polyoxyethylene chains, which could decrease penetration into the keratin matrix. Moreover, the experiment was conducted in an aqueous medium, which could result in the formation of large micelles. This effect could modify the composition of the nail and decrease penetration of hydrophobic molecules, like amorolfine hydrochloride [26]. 

Application of thioglycolic acid or urea (10% (v/v)) caused coiling and breaking of the hoof lamella. A similar effect on the nails has been described when thioglycolic acid and urea were applied [27]. Incorporation of 0.5% salicylic acid into the liquid mixtures of lacquers resulted in visible precipitates and reduced film-forming ability, delayed drying, and poor spreading characteristics. Preformulation studies demonstrated acceptable compatibility of thioglycolic acid and urea with used nail lacquer formulas. The above results could be referred to etching and disulfide bonds disrupting effect, which can be caused by increased internal surface area inside the nail. The delayed release could be attributed to the swelling of the nail and limited paths for diffusion of amorolfine hydrochloride. 

### 3.2. Formulation and Evaluation of Nail Lacquers

Formulation studies demonstrated that the increase of the quantity of film-forming polymer Eudragit E100 to concentrations higher than 20% resulted in the yellowish coloring of the formulation, increased viscosity and prolonged drying times. Nail lacquers were formulated with a minimum (5%) and maximum (20%) Eudragit E100 concentrations, resulting in flowy and efficiently drying formulations and their quantitative compositions are presented in Table 1. 

The testing of resulting lacquer films demonstrated, that resistance to water increased with the increase of the quantity of film-forming polymer in the formulation. The film stayed attached to the testing surface up to 72 h when Eudragit E100 concentration was 5% in the liquid nail lacquer formulation, and up to 120 h when Eudragit E100 concentration was increased to 20%. 

The addition of thioglycolic acid or urea prolonged drying times twofold if compared to enhancer free formulation. The specific odor of the film containing thioglycolic acid was identified. 

The in vitro release profiles of amorolfine (Table 2) from lacquer films N1 and N2 were not significantly affected by the quantity of film-forming polymer. The incorporation of penetration enhancers demonstrated a different effect on amorolfine hydrochloride release.

Statistically significant (*p* < 0.05) increase of amorolfine hydrochloride release was determined when thioglycolic acid was included in the formulation if compared to formulations containing no penetration enhancer. Opposite effect on amorolfine release was determined when urea was added to the formulations. The release of the drug substance decreased statistically significantly (*p* < 0.05) if compared to enhancer-free formulations. This effect can be related to possible interactions between drug, polymer, and penetration enhancer. Moreover, it was previously demonstrated, that Eudragit E100 can interact and destabilize natural membranes, displaying a strong amphipathic activity [28]. With reference to the highest quantities of amorolfine released, formulations containing 20% of film-forming polymer were selected for further transungual studies. 

### 3.3. Amorolfine Hydrochloride Transungual Delivery

Transungual penetration studies of amorolfine hydrochloride were performed using non-laser treated and laser affected human nail clippings. Three formulations (N2, N4, and N6) were tested, and the results of penetration studies were evaluated. 

Tested formulations (0.006–0.01g) were applied on nail clippings (approx. 0.003 g). The thickness and weight changes of the nail clippings were determined (Table 3). Up to 35% increase in the weight of nail clippings during the experimental procedure was determined, thus indicating the possibility of changing barrier properties of the nail matrix. The significant weight increase was not followed by an appropriate increase of the thickness (maximum increase up to 6.25%), therefore it could be expected that the internal network of keratin was not significantly affected. 

Transungual penetration can be affected by the physicochemical properties of the drug molecule. It has been demonstrated, that smaller molecules diffuse better through the pores of the keratin network [7]. Amorolfine hydrochloride should penetrate relatively easy into nail plate, as its molecular weight of 353.975 g/mol is considered medium.

**Figure 2:**
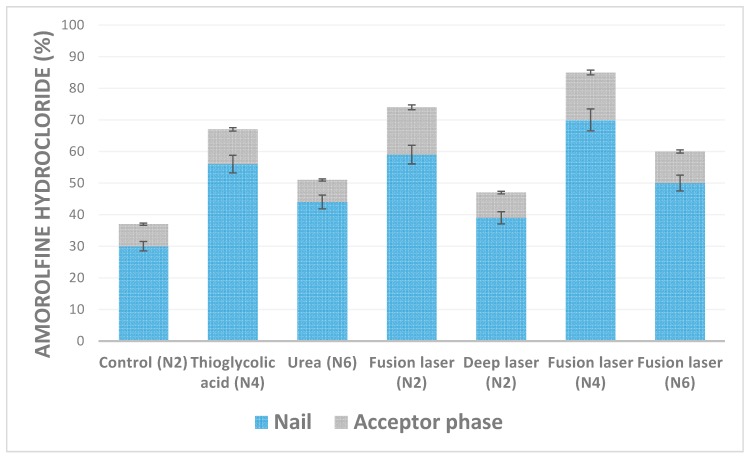
Chemical and physical enhancement of penetration of amorolfine hydrochloride after application nail lacquers (n = 2).

In this study, we used healthy volunteers nail clippings. The use of the nails clippings from the same few volunteers guaranteed a controlled variation of nail thickness. It has been demonstrated, that the drug permeability could be considerably influenced by the nail thickness [16]. During transungual delivery studies, chemical and physical enhancement methods were used separately and combined. These findings are presented in Figure 2. 

**Figure 3:**
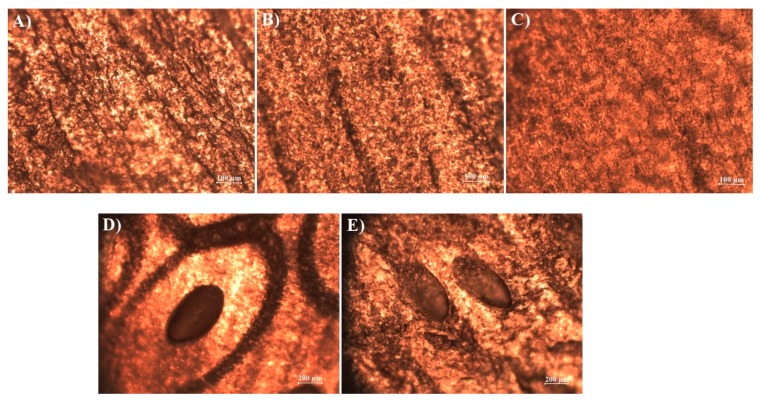
Surface changes in nail clippings throughout the penetration experiment: (**a**) natural structure nail surface; (**b**) nail after 24 h in 5% urea solution; (**c**) nail after 24 h in 5% thioglycolic acid solution; (**d**) laser-affected nail surface; (**e**) laser-affected nail surface after 24 h application of nail lacquer with 5% thioglycolic acid as chemical enhancer.

Thioglycolic acid and “fusion” laser energy had a comparable enhancement effect on the accumulation of amorolfine hydrochloride in nails, which increased by 56% and 59%, respectively. Up to 70% of the applied dose of amorolfine hydrochloride accumulated in the nails when these two methods were combined. However, no synergistic effect—of laser pretreatment and thioglycolic acid presence in the lacquer—on amorolfine hydrochloride permeation was established, which can be explained by their different mode of action on the nail. Thioglycolic acid has limited access to deeper nail layers and, thus, the nail maintains its barrier function with respect to the applied drug. A similar amount of amorolfine hydrochloride was determined to be in the acceptor phase when only “fusion” laser pretreatment was performed. Laser-induced disruption of the nail created microchannels, shortened the diffusion pathways, and facilitated drug delivery into deeper layers of the nail. These findings were visualized by microscopy (Figure 3)—thioglycolic acid etching effect was observed. This visualization could explain higher accumulation rates as the contact area was bigger if compared to the application of individual enhancement methods. 

There were no significant differences between urea and “deep” laser energy enhancement effect. These methods increased the accumulation of amorolfine hydrochloride in nails, but penetration rates were the same as in the control group. As was indicated earlier, the use of single keratolytic agent results in little or no transungual permeation enhancement [7]. Also, no increase in the enhancement effect was observed when laser energy power was increased. 

Summarizing the results of transungual penetration studies, it should be emphasized that the determined concentrations of amorolfine hydrochloride in the tested biological membranes and in the acceptor phase exceeded MIC levels [9]. Thus, it could be expected that tested formulations should be effective in onychomycosis treatment even when considering the necessity of additional validation of the suitability of applied methods when designing optimal ungual formulations. 

## 4. Conclusions

Attempts to produce nail lacquer formulation satisfactorily combining acceptable applicability and adequate biopharmaceutical characteristics presents a significant challenge. This may be mainly attributed to the limited list of excipients available for lacquer formulation and varying approaches in the biopharmaceutical testing of dosage. The current study demonstrated that the increase of Eudragit E100 polymer quantity from 5% to 20% in the formulation resulted in increased release of amorolfine hydrochloride from lacquer film, up to 20% in 4 h, resulting in the release of 76.3% of amorolfine quantity available in lacquer film.

The current study demonstrated that more efficient amorolfine hydrochloride transungual delivery could be achieved when physical and chemical enhancers were used in combination. Laser pretreatment affected the structure of the nail. A significant increase in drug accumulation was achieved when laser pretreatment was combined with thioglycolic acid chemical enhancement effect. However, no synergistic effect of laser pretreatment and thioglycolic acid presence in the lacquer on amorolfine hydrochloride permeation was established. Overall, this novel combination of enhancers could provide new prospects in onychomycosis treatment. 

## Figures and Tables

**Table 1:** Compositions of formulated experimental lacquers with amorolfine hydrochloride.

Formulation Code	N1	N2	N3	N4	N5	N6
Eudragit E100	5	20	5	20	5	20
Ethanol (96%)	85	70	80	65	80	65
Transcutol P	5	5	5	5	5	5
Thioglycolic acid	-	-	5	5	-	-
Urea	-	-	-	-	5	5
Amorolfine hydrochloride	5	5	5	5	5	5

**Table 2:** Amorolfine hydrochloride release (%) from experimental lacquers.

Formulation Code		Released Amount, %					
		N1	N2	N3	N4	N5	N6
Time (h)	1	33.2 ± 0.1	35.1 ± 1.2	40.4 ± 0.9	43.4 ± 1.1	18.5 ± 0.6	28.1 ± 0.4
	2	44.1 ± 0.4	48.5 ± 1.1	49.4 ± 0.7	56.2 ± 1	25.5 ± 0.7	29.3 ± 0.6
	3	55.2 ± 0.3	57.3 ± 0.8	54.7 ± 0.4	64.1 ± 0.8	36.7 ± 0.75	40.8 ± 0.8
	4	62.4 ± 0.7	69.2 ± 0.7	68.3 ± 0.8	76.3 ± 0.7	39 ± 0.78	48.5 ± 0.7

**Table 3:** Non-laser treated nail clippings thickness and weight changes during penetration studies.

Nail Clippings	Thickness (µm)			Weight (g)		
	N2	N4	N6	N2	N4	N6
Initial	810 ± 1.1	800 ± 0.8	800 ± 0.7	0.0108 ± 0.002	0.0128 ± 0.002	0.0109 ± 0.001
After penetration study	850 ± 1.5	820 ± 1.2	850 ± 1.1	0.0140 ± 0.001	0.0175 ± 0.001	0.0147 ± 0.002
Change (%) compared to initial	+4.9	+2.5	+6.25	+29	+27	+35
After drying	810 ± 1.1	800 ± 0.8	800 ± 0.7	0.0106 ± 0.001	0.0124 ± 0.002	0.0107 ± 0.001
Change (%) compared to initial	0	0	0	−1.8	−3.2	−1.8
